# Current situation in radiation oncology residency—Results of a national survey performed by the working group Young DEGRO of the German Society of Radiation Oncology

**DOI:** 10.1007/s00066-023-02137-y

**Published:** 2023-08-29

**Authors:** Daniel F. Fleischmann, Marcel Büttner, Michael Oertel, Maria Waltenberger, Christoph Süß, Sonia Ziegler, Lukas Käsmann, Danny Jazmati, Annemarie Schröder, Matthias Mäurer, Philipp Linde

**Affiliations:** 1 Department of Radiation Oncology, University Hospital, LMU Munich, Munich, Germany; 2 Partner site Munich, German Cancer Consortium (DKTK), Munich, Germany; 3 German Cancer Research Center (DKFZ), Heidelberg, Germany; 4 Department of Radiation Oncology, University Hospital Muenster, Muenster, Germany; 5 Department of Radiation Oncology, Klinikum rechts der Isar, Technical University of Munich (TUM), Munich, Germany; 6 Department of Radiotherapy, University of Regensburg, Regensburg, Germany; 7 Klinik für Strahlentherapie und Radioonkologie, Universitätsmedizin Göttingen, Göttingen, Germany; 8 Department of Radiation Oncology, University Hospital Dusseldorf, Medical Faculty, Heinrich Heine University of Dusseldorf, Dusseldorf, Germany; 9 Department of Radiotherapy and Radiation Oncology, University Medical Centre Rostock, Rostock, Germany; 10 Department of Radiotherapy and Radiation Oncology, University Hospital, Friedrich-Schiller-University, Jena, Germany; 11 Department of Radiation Oncology, Cyberknife and Radiation Therapy, Faculty of Medicine and University Hospital of Cologne, University of Cologne, Cologne, Germany; 12 Center for Integrated Oncology (CIO), University Hospital of Cologne, Faculty of Medicine, University of Cologne, Cologne, Germany

**Keywords:** Further education, Radiation oncology, Teaching, Residency, Curriculum, Training, Feedback, Supervision

## Abstract

**Background:**

The aim of this study was to assess the current status of the radiation oncology (RO) residency programs in Germany. For this, RO residents and RO specialists were surveyed regarding the current situation of the RO residency training and the working conditions in Germany.

**Methods:**

The Continuing Education Section of the Young DEGRO (yDEGRO) Working Group of the German Society of Radiation Oncology (DEGRO) developed a survey to assess (1) the overall satisfaction, learning objectives, and teaching methods used during training; and (2) the perception of the importance of specific disease patterns in RO training. Open-ended questions were also asked to elicit opinions on areas for improvement. From 21 November to 27 December 2022, RO residents registered with DEGRO and/or in the working group yDEGRO were invited to participate anonymously in an online questionnaire.

**Results:**

Overall, 97 participants completed the survey, including 65 RO residents (67%) and 32 RO specialists (33%); 66 (68%) of the respondents reported being employed in the university setting, 23 (23.7%) in the non-university setting, and 8 (8.3%) in private practice. Within the training, heterogeneity was found in the teaching methods used. In terms of knowledge transfer, the greatest importance was accorded to annual continuing education discussions with the head of the residency training (92.8%), participation in tumor boards (85.6%), written training concepts (81.4%), and evaluations at the beginning (76.3%) and end of a rotation (80.4%). The arithmetic mean of satisfaction with specialist training was 6/10 points (SD: 1.99); 88.7% of respondents would like to see a nationally uniform and mandatory curriculum in RO residency training.

**Conclusion:**

The study provides suggestions for improving RO medical training in Germany: further development of accompanying education and training programs in cooperation with professional associations, e.g., the DEGRO, structured feedback, and supervision.

## Introduction

Quality of residency training and education is important in order to secure the next generation of radiation oncology (RO) specialists and to remain competitive with other disciplines. In the era of personalized oncology treatment regimens and highly advanced RO treatment techniques, the complexity of RO is steadily increasing [1]. This requires appropriate residency training programs to ensure that the residents acquire all the knowledge and skills that they need for their daily work in RO.

In recent years, the Young DEGRO (yDEGRO) Working Group of the German Society of Radiation Oncology (DEGRO) has conducted a number of surveys on the status quo of RO training [2–4]. The need for restructured curricula has been expressed several times. The webinars on residency training were successfully implemented in 2020/2021 in a collaborative project between DEGRO and yDEGRO. Feedback from participants of the webinars, discussions with the DEGRO board during the development process of the webinars, and a suspected impact of the COVID-19 pandemic on the structure of continuing education prompted the continuing education section of the yDEGRO working group to conduct a survey on the current situation of RO residency training in Germany.

In this survey, we aimed to access knowledge about teaching events available in residency training sites in Germany, effective ways of knowledge transfer throughout residency training, and current knowledge of RO residents and specialists on the major cancer entities. On the basis of the findings, we measured the need for continuing medical education and the necessity of financial reimbursement for attendance at continuing medical education events and congresses. Finally, we assessed the need for short-term clinical visit programs and clinical rotations in the residency programs in Germany, the preparedness for board examination through residency, and the need for a standardized RO residency training curriculum in Germany.

## Methods

### Study design and procedures

A questionnaire was designed by the working group of the yDEGRO in a peer-review process to assess the extent and topics of RO teaching. The anonymous online survey using a protected document was sent via an open-source software (Survey Online®, Enuvo Inc., Switzerland) to ROs working in RT departments and training institutions.

Invitations to participate in the study were sent out via the institutions’ professional mailing lists (DEGRO, yDEGRO; 800 email addresses), after a letter had been sent to each RO department head informing them about the project and asking them to distribute the questionnaire in their departments. In addition to the email invitation, participation in the survey was promoted at each site by local yDEGRO representatives and during the residency training webinar in December 2022. The survey was open for 1 month, from 21 November to 27 December 2022.

### Survey description

The first part of the survey covered the sociodemographic data of the respondents (four items). The second part consisted of questions about the type and design (structural, methodological, temporal) of the teaching formats at the respective institution (seven items). The next 12 items asked about the possibilities of participation, time resources (e.g., time off clinical duties), and financing of short-term clinical visit programs and continuing professional development events (continuing medical education events, congresses). Ten items contained complexes of questions on the form of knowledge transfer, the competencies required to achieve specialist status, the assessment of one’s own level of knowledge, and the significance according to the 11 most important disease entities, as well as knowledge of the yDEGRO short-term clinical visit programs. Relevance and appropriateness were assessed for all of these items. The last two questions addressed the general satisfaction with the training and whether there was a desire for a universal, location-independent curriculum for further RO training in Germany.

Participants were also asked to rate the relevance of the topics taught in their training curriculum and their level of knowledge of these topics using Likert scales. One item addressed the dimensions of diversity (“equal access with equal resources for all medical professionals”). Finally, through open-ended questions respondents were asked to suggest ways to improve the training program. In total, the survey consisted of 37 questions (see Additional file 1).

### Data analysis and statistical methods

Classic descriptive statistics were used to describe the data set. In particular, frequencies and percentages are given for qualitative variables, while standard deviation, median and interquartile range (Q25–75) are used to summarize quantitative variables due to non-normality. Statistical analysis was performed with the SPSS Statistics software package version 29 (IBM, Armonk, NY, USA) for descriptive statistics such as the mean, median, range, and standard deviation.

For the analysis of the open-ended questions, we used a combined approach with direct content analysis according to Mayring by two reviewers (DF, PL) for each individual free-text response, followed by a summative content analysis [12]. In this way, the identification process and the creation of coding categories were structured in an objectifiable way. The identified categories were examined for consistency with the content of the Canadian Medical Education Directives for Specialists (CanMEDS) Roles in its latest version [5]. Each of these directives is based on core competencies that must be applied in that role.

### Ethical standards

The survey was conducted in accordance with the ethical standards of the Institutional Research Committee and the Helsinki Declaration of 1964 and its subsequent amendments, or comparable ethical standards. The questionnaire was provided on the basis of and in accordance with the regulations of DEGRO’s data protection conditions [13]. According to the Ethics Committee of the Medical Faculty of LMU Munich, prospectively anonymized questionnaires do not require ethical consultation.

## Results

### Study participants

In total, 97 participants completed the survey and were included in the study. Of these, 49 (50.5%) were male, 47 (48.5%) were female, and one (1.0%) identified as diverse. The median age was 34 years (range: 25–57 years). Current place of employment was university hospital, non-university hospital, and private practice for 66 (68.0%), 23 (23.7%), and eight (8.3%) respondents, respectively. The current place of employment had permission to provide residency training for the complete vs. only part of the residency training period in 89 (91.8%) vs. eight (8.2%) cases, respectively.

Overall, 65 (67.0%) of the participants in this survey were residents and 32 (33.0%) were board-certified radiation oncologists. Of the residents, 29 (44.6%) were in their first to third year, 27 (41.5%) in their fourth or fifth year, and nine (13.9%) in their sixth or later year of residency training. Further information on the characteristics of the participants is shown in Table 1.

**Table 1:** Characteristics of residents and radiation oncology specialists

Characteristic	All participants (*N* = 97)	Residents (*N* = 65)	Radiation oncology specialists (*N* = 32)
*Gender*			
Male	49 (50.5%)	30 (46.2%)	19 (59.4%)
Female	47 (48.5%)	34 (52.3%)	13 (40.6%)
Diverse	1 (1.0%)	1 (1.5%)	0
*Median age in years (range)*	34 (25–57)	32 (25–46)	42 (31–57)
*Year of residency*			
Year 1	–	5 (7.7%)	–
Year 2		13 (20.0%)	
Year 3		11 (16.9%)	
Year 4		16 (24.6%)	
Year 5		11 (16.9%)	
Year 6 or higher		9 (13.9%)	
*Workplace*			
University hospital	66 (68.0%)	49 (75.4%)	17 (53.1%)
Non-university hospital	23 (23.7%)	13 (20.0%)	10 (31.3%)
Private practice	8 (8.3%)	3 (4.6%)	5 (15.6%)
*Authorization of continuing education at workplace (Weiterbildungsbefugnis)*			
Authorization for complete time of residency	89 (91.8%)	61 (93.8%)	28 (87.5%)
Authorization for only part of the time of residency	8 (8.2%)	4 (6.2%)	4 (12.5%)

### Radiation oncology educational events available at the workplace

Radiation oncology educational events were offered as in-person only, virtual only, and both in-person and virtual teaching events at 35 (36.1%), 10 (10.3%), and 45 (46.4%) sites, respectively. Seven participants reported that at their residency training sites (7.2%) no teaching events were offered.

Teaching events were offered more than once a week, once a week, once a month, once per quarter, or never in 23 (23.7%), 49 (50.5%), 14 (14.4%), six (6.2%), and five (5.2%) cases, respectively. Preparation of the teaching events was performed mostly by residents, with or without the supervision of RO specialists or senior consultants, RO specialists, senior consultants, and chief physicians in 14 (14.4%), 47 (48.5%), eight (8.2%), 21 (21.7%), and seven (7.2%) cases, respectively. The most common types of RO teaching formats available on site were educational series on specific topics (71.1%), journal clubs (60.8%), and morbidity and mortality conferences (49.5%). Fewer than half of the sites offered detailed case and indication discussions (34.0%), interdisciplinary continuing education events, e.g., together with surgery or internal medicine colleagues (32.0%), presentations of case reports of interest (24.7%), or detailed case presentation with a didactic focus (13.4%).

### Days off from clinic for continuing medical education events

Days off from the clinic for continuing medical education events were reported to be more than 5 days, 4–5 days, 1–3 days, none, or not determinable in 14 (14.4%), 40 (41.3%), 30 (30.9%), one (1.0%), and 12 (12.4%) cases, respectively. More than half of the participants (50.5%) wished to have the opportunity to have 5 days off or more from the clinic for continuing medical education events. Participants reported the same support concerning education regardless of gender, religion, or sexual orientation in 78 (80.4%) cases, while 14 (14.4%) participants were unsure about this question and five (5.2%) reported differences.

### Forms of knowledge transfer present and warranted in residency training

**Fig. 1:**
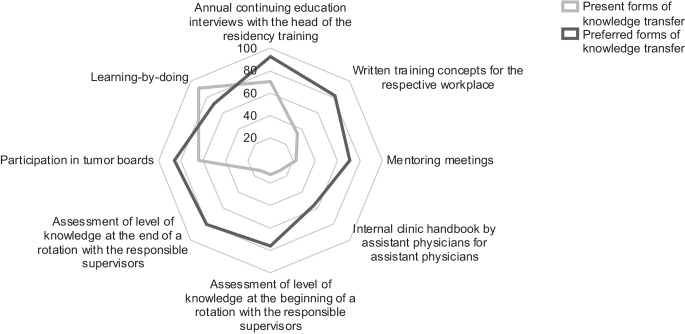
Forms of knowledge transfer present vs. forms of knowledge transfer warranted

The most important forms of knowledge transfer in residency training that participants wanted to be present at every residency site were (1) annual continuing education interviews with the head of the residency training (92.8%), (2) participation in tumor boards (85.6%), (3) written training concepts for the respective workplace (81.4%), (4) assessment of the level of knowledge at the beginning of a rotation (76.3%) as well as (5) assessment at the end of a rotation with the responsible supervisors (80.4%), (6) mentoring interviews (71.1%), (7) learning-by-doing (71.1%), and (8) in-house manuals by residents for residents (55.7%). The most present forms of knowledge transfer were learning by doing (90.7%), the annual continuing education interviews with the head of the residency training (70.1%), participation in tumor boards (63.9%), as well as written training concepts for the respective workplace (34.0%), while the other forms of knowledge transfer were present in less than one quarter of the cases. Representation in tumor boards was performed mostly by senior consultants, chief physicians, and RO specialists without senior consultant status and less commonly by residents under the guidance of a board-certified physician (88.7%, 55.7%, 51.5%, and 15.5%, respectively). The aforementioned forms of knowledge transfer are shown in Fig. 1.

### Knowledge about key oncological topics and importance of continuing education

While knowledge about the most commonly treated entities such as breast cancer, prostate cancer, lung tumors, colorectal cancer, head and neck cancer, brain metastases, and primary brain tumors was rated with a median score of 7 out of 10 or higher, knowledge about less commonly treated entities such as gynecological tumors of the pelvis, lymphomas, skin tumors, and malignancies of the urinary tract was rated with a median score of 6 out of 10 or lower. The importance of continuing medical education, such as webinars, was reported to be a median of 8 out of 10 or higher for all of the aforementioned entities. The topics of the academic curriculum of the DEGRO Academy covering all major entities were known by only 35 (36.1%) participants, while 62 (63.9%) were not familiar with these topics. Further details of the participants’ knowledge of key oncological topics and their assessment of the importance of continuing medical education on these topics are shown in Table 2.

**Table 2:** Self-assessed knowledge vs. need for further continuing medical education by cancer entities

Cancer entity	Self-assessed knowledge (*N* = 97)	Reported need of further continuing medical education (*N* = 97)
	Median (IQR)	Median (IQR)
Breast cancer	8 (6–9)	10 (8–10)
Prostate cancer	8 (6.5–9)	10 (8–10)
Colorectal cancer	8 (5.5–8)	10 (8–10)
Gynecological cancers (besides breast cancer)	6 (4–8)	9 (8–10)
Head and neck cancer	7 (6–9)	10 (8–10)
Lymphomas	4 (3–7)	9 (6.5–10)
Skin tumors	5 (3–7)	8 (6–10)
Cancers of the urinary organs	6 (4–7)	8 (6–10)
Brain metastasis	9 (8–10)	10 (7–10)
Primary brain tumors	8 (7–9)	9 (8–10)

*IQR* interquartile range

### Reimbursement for continuing medical education

When asked about the percentage of residents who were reimbursed for costs of continuing medical education, for events held in Germany, a majority of 58 respondents reported that all residents were reimbursed (59.8%). For educational events held in or outside the EU, only 21 (21.6%) and 12 (12.4%) institutions, respectively, offered reimbursement for all residents. In particular, for educational events held in Germany and the EU, respondents wished to have a higher level of reimbursement, with 80 (82.5%) and 49 (50.5%) respondents, respectively, wishing to have reimbursement for all residents for these events. Complete reimbursement for continuing medical education was reported by 48 participants (49.5%) only, while 74 (76.3%) expressed the need for complete reimbursement. Mandatory courses during residency, such as radiation protection courses, were fully or partially reimbursed for 81 (83.5%) and 12 (12.4%) residents, respectively.

### Reimbursement for congresses

For congresses held in Germany, 55 (56.7%) respondents reported that all residents were reimbursed. For congresses held in the EU or outside the EU, only 26 (26.8%) and 15 (15.5%) sites, respectively, offered reimbursement for all residents. Again, especially for congresses held in Germany and the EU, respondents wanted to have a higher level of reimbursement, with 68 (70.1%) and 45 (46.6%) respondents, respectively, wanting reimbursement for all residents for these events. Reimbursement for congresses was in 28 (28.9%) cases linked to the condition of giving a lecture or presenting a poster, or reserved exclusively for giving a lecture in 10 (10.3%) cases. Full reimbursement for congresses was reported by 43 (44.3%) participants only, while 72 (74.2%) expressed the need for full reimbursement.

### Short-term clinical visit programs and clinical rotations

When asked about opportunities to participate in short-term clinical visit programs at their site, 22 (22.7%) respondents indicated that they would be able to participate in the yDEGRO short-term clinical visit program, and eight (8.2%) indicated that they would be able to participate in the yDEGRO brachytherapy short-term clinical visit program.

A total of 68 respondents (70.1%) stated that the opportunity of short-term clinical visit programs was unknown to them. Participation in short-term clinical visit programs and clinical rotations was not mandatory for the majority of participants (*n* = 80; 82.5%). Mandatory hospitalizations or clinical rotations to another RO site, to another medical specialty at the same site, to another medical specialty at a different site was present for eight (8.2%) participants, six (6.2%) participants, and one (1.0%) participant only.

When asked on the number of residents who participated in a short-term clinical visit program or a clinical rotation at least once during their residency, the most common response was none (56.7%) and only a few (23.7%), meaning less than one in four.

### Suggestions to improve the training program—necessary competencies for all areas of medical practice: qualitative analysis

At the end of the survey, 27 participants answered the open-ended question about their wishes for radiotherapy residency training in Germany. More time for one’s own continuing education, the desire for standardized basic knowledge of RO, learning content on radiologic imaging and reporting, and mandatory rotations to (1) other RO clinics and (2) institutions outside the specialty were most frequently mentioned. The above categories were matched with the seven professional competencies defined in the CanMEDS model for consistency. In this process, the wishes could be brought into line with the following competencies:

- Scholar (continuing education, teaching, and research)
- Professional (ethical standard and excellence)
- Collaborator (collaboration with other health professional)
- Medical expert as the integrating role (theoretical and practical knowledge)

**Fig. 2:**
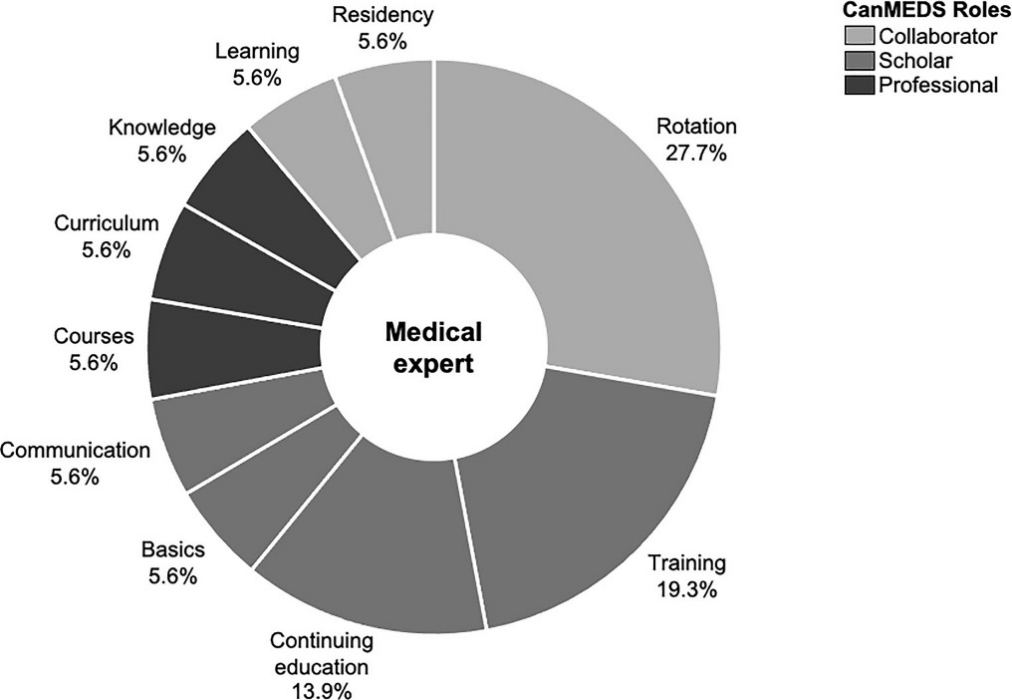
Wishes for improvement in radiation oncology residency sorted by CanMEDS roles. *CanMEDS* Canadian Medical Education Directives for Specialists

Based on our content analysis, CanMEDS roles Leader (management of human and technical resources), Health Advocate, and Communicator (appropriate and effective communication) could not be aligned (see Fig. 2).

### Preparedness for board examination

When asked about their perception on their preparedness for the RO board examination, 56 (57.7%) participants stated that they knew what was expected from them, while 41 (42.3) stated they did not. Satisfaction with preparation for the board examination at the current training site was reported with a median score of 7 out of 10 points (standard deviation 2.23 points). Overall satisfaction with residency training was reported with a median score of 6 out of 10 points (standard deviation 1.99 points). A majority of 88.7% of respondents stated that they would like the young DEGRO to work towards a uniform, site-independent continuing education curriculum for residency training in Germany.

## Discussion

Due to the increasing incidence of cancer, there is a growing need for well-trained radiation oncologists, and concerns were raised already a decade ago both in the United States [6] and in the EU [7] about the ability to keep up with the growing demand. In recruiting new residents, one of the biggest challenges is that the number of medical students remains constant, while the number of cancer patients is increasing due to an aging population. The projected increase in cancer in our aging population requires active support of both the current and the next generation of radiation oncologists [8]. In Germany, securing the next generation of radiation oncologists must be a stated goal of the DEGRO for the near future, both in light of the aforementioned demographic trends of the population and of DEGRO members.

According to the DEGRO office, the average age of physicians registered in the DEGRO in 2022 was 49 years for women and 53 years for men. The goal of the medical society and the yDEGRO must therefore be to get as many medical students as possible interested in RO during their medical training, to then retain them in the specialty, and to offer state-of-the-art residency training and continuing education [9].

The impending shortage of radiation oncologists in Germany was already addressed more than 15 years ago in a survey by the DEGRO [10]. Here, the residents surveyed expressed a high level of overall satisfaction with the training itself and also indicated that they would choose RO as a specialty again [10]. This highlights the importance of ensuring that medical students are properly introduced to RO, as the challenge appears to be in introducing aspiring radiation oncologists to the field, not to keep them in it. A fixed curriculum was only available in 2009 at slightly more than half of the sites (55.8%; [10]). In addition, bottlenecks in the rotations of the individual workplaces were reported by 38.9% of the respondents. A demand was expressed for continuous supervision by the training officers and better integration with radiology and internal oncology [10], which was also expressed in the current survey.

The subsequent 2016 survey of the yDEGRO on the training situation of residents continued to show a high level of satisfaction with the residency in Germany with good working conditions overall [3]. As in the current survey, a need for improvement in education was identified, especially for entities that are treated less frequently in RO. In particular, knowledge of pediatric and ophthalmologic malignancies as well as lymphomas and leukemias was reported to be the lowest by respondents [3]. A survey of U.S. residents also revealed a need for improvement in knowledge of pediatric malignancies and lymphomas [11]. Consistent with these findings, respondents of the current survey expressed a strong interest in additional educational opportunities, such as webinars, not only for the common entities treated with radiotherapy, but also for the less frequent ones.

In a 2022 survey on brachytherapy education during residency, 68% of the residents surveyed indicated that no brachytherapy curriculum existed at their institution [12]. In addition, 47% of respondents reported a lack of didactically prepared teaching to be an obstacle in their brachytherapy training [12]. Therefore, the authors concluded that the implementation of a brachytherapy curriculum and the presence of well-trained instructors were of utmost importance [12]. Here, the residents could additionally take advantage of the short-term clinical visit programs offered by the yDEGRO’s *Continuing Education *section. The current survey shows that the level of awareness of this program needs further development, as these programs were not known to a high proportion of RO residents in Germany, although these programs have regularly been promoted at the DEGRO annual meetings and on yDEGRO’s webpages.

In a qualitative study conducted in 2021, in which medical students were asked for suggestions to improve the teaching of RO, students pointed out that RO is still underrepresented in medical studies in Germany, despite its importance in the treatment of cancer [13]. Medical education in Germany is currently undergoing a longitudinal restructuring in the sense of competency-based learning [9]. This offers great opportunities to anchor the outstanding role of RO in cancer treatment in medical education longitudinally and to ensure an adequate number of committed young professionals in RO. Previous teaching concepts have shown that RO content can be taught to medical students already in the preclinical phase of their studies to familiarize and get them interested in RO at an early stage [4]. Modern teaching formats, including e‑learning that enables the flipped classroom, have recently been shown to improve RO education [14] and may be a key to attracting more medical students to RO.

Despite the tremendous efforts of both DEGRO [15] and European Society for Therapeutic Radiology and Oncology (ESTRO) [16, 17] to integrate RO curricula into practice, respondents to this survey expressed a need for further integration of these RO curricula at their residency training site. A large majority of respondents of the current survey indicated that they would like to see the yDEGRO work toward a uniform, site-independent continuing education curriculum for residency training in Germany.

In 2015, the latest version of the Canadian Medical Education Directives for Specialists (CanMEDS 2015) was published, setting out the updated framework for training and the educational continuum for physicians from entry into postgraduate medical education, i.e., residency training, to the time of retirement [5]. CanMEDS describes seven overlapping domains of competence [5]. This framework already forms the basis for the ESTRO core curriculum in its current version [18]. The wish of the participants of the current survey for a uniform German RO core curriculum for RO residency training should take into account the ESTRO guidelines and the CanMEDS roles in a conceivable implementation. The definition of an RO specific competency set provides a basis for the development of tools to support learning and teaching for all RO trainees, including medical students. It can be assumed that learning all CanMEDS competencies will lend itself well to interprofessional educational opportunities involving the entire RO team. In this regard, there are already several initiatives underway, which apply these competencies to RO (medical) education.

In terms of competencies, Dapper et al. [14] and Linde et al. [13] were able to establish useful objectives for standardized teaching in RO education as well as alignments in what medical students expect from specific RO education. Their findings are consistent with our data and are also consistent with the respondents’ wishes. For example, the desire for rotations in radiologic diagnostics is considered particularly valuable by prospective radiation oncologists and is desired as an integral part of residency training.

Furthermore, this demand for a well-founded training in the assessment of radiological image data sets corresponds to both the consensus specifications in the curriculum of the DEGRO *Academy* working group [15] as well as to the guidelines for specialist training as a radiation oncologist of the German Medical Association (*Bundesärztekammer*; [19]). Consideration of modern university teaching and an alignment and subsequent adjustment of the training of medical students and physicians could be a guiding goal to ensure the focus for the future and the assurance of RO care in Germany [20].

In contrast to the reported satisfaction of the participants in the present survey with an average score of 6.51 on a Likert scale of 1–10, the Monitor Report of the Marburger Bund, published in August 2022, revealed a growing dissatisfaction of physicians with the professional situation [21]. A quarter of the 8464 employed physicians surveyed are even considering a change of profession [21]. Increasing workloads and inadequate staffing levels were reported to be the primary reasons for this dissatisfaction [21]. The task of the healthcare system and those involved in the structure must actually be to keep everyone in the medical profession as much as possible. This is especially true for RO with its increasing demand for specialists, even though the current survey shows that the current situation regarding job satisfaction in RO seems to be above average, when looking at the aforementioned Monitor Report of the Marburger Bund [21].

Mentoring discussions and evaluations at the beginning and end of a rotation with the responsible supervisors were desired by more than two thirds of respondents of the current survey, but were present in less than one quarter of residency programs. Consistent with the responses in our survey, a recent literature review found great opportunities for RO resident career development through mentorship initiatives [22]. A large survey of mentoring programs in the United States found that residents who had participated in a mentoring program were more satisfied with their mentoring experience, but only large training institutions offered these programs, leaving a critical unmet need according to the authors [23].

Given the possibility of sampling bias, the authors believe that the achieved response rate of 17% is a highly satisfactory representation of physicians. A possible limitation of the survey, that should be mentioned, is that physicians from faculties that are more involved in training and teaching may have responded to the survey at a higher rate. This could lead to a bias in the results and an underestimation of potential problems in teaching RO, too.

The survey results should be viewed as a point-in-time observation. It provides comparability between the different training sites in an anonymized form, offers suggestions for further improvements in the teaching of radiation oncology (RO) and the implementation of state-of-the-art teaching concepts.

## Conclusion

The current survey supplies concrete and leading ideas for improving RO medical training in Germany. Suggestions for improvement include further development of accompanying education and training programs in cooperation with professional associations, e.g., the DEGRO, as well as the wish for structured feedback and supervision. The design and definition of a nationwide uniform curriculum with key qualifications and knowledge of the RO specialist status should be one of the core visions for the future of those involved in the training of physicians.

## Appendix

Survey, full text. Literal translation from German to English.

The original questionnaire was distributed in German.

### Young DEGRO *continuing education* survey: knowledge acquisition and transfer in continuing education

1. This is an anonymous survey. No personal information about you is stored in the survey responses. The general data protection conditions of the German Society for Radiation Oncology (DEGRO)—https://www.degro.org/degro/datenschutz/.

Answer (A.): Yes, I have read and accept the privacy policy.

2. Gender. *Drop down menu.*

A.: Female, male, divers, other.

3. Year of residency training. *Drop down menu.*

A.: Year 1, 2, 3, 4, 5, Already a consultant/specialist.

4. Age statement

A.: Single mention or negative answer with the post 999.

5. Where are you working? *Drop down menu.*

A.: Medical/private practice. University. Non-university hospital.

6. Are internal teaching formats currently available at your site? *Drop down menu.*

A.: Yes, only in person. Yes, virtual only. Yes, in person and virtually. No.

7. On average, how many hours per week do in-house teaching formats account for at your site? *Drop down menu.*

A.: More often than 1 × per week. 1 × per week. 1 × per month. 1 × per quarter. 1 × per year. Never.

8. Who prepares the internal courses most frequently? Please arrange from mostly to rarely (1 to 5). *Drop down menu with a range from 1 to 5.*

A.: Resident physicians. Residents under the guidance of senior physicians. Specialists.

Senior physicians. Chief physicians.

9. For how many years is the institution where you do work certified for residency training? *Drop down menu.*

A.: Full. In parts. Not at all.

10. What form of teaching formats are available in-house? *Drop down menu.*

A.: Journal Club (classical format with 1 paper). Advanced training series (topic-specific).

Interdisciplinary training (e.g. with surgery or internal medicine). Medical-didactic detailed case presentation, e.g. 30 to 45 min. Detailed discussion of RO indications for all staff as part of case presentations. Short presentation of an interesting case report. Morbidity and mortality conferences (M&M). Other (free text).

11. What form of teaching formats are available in-house? Please arrange from mostly to rarely (1 to 7). *Drop down menu with a range from 1 to 7.*

A.: Journal Club (classical format with 1 paper). Advanced training series (topic-specific).

Interdisciplinary training (e.g. with surgery or internal medicine). Medical-didactic detailed case presentation, e.g. 30 to 45 min. Detailed discussion of RO indications for all staff as part of case presentations. Short presentation of an interesting case report. Morbidity and mortality conferences (M&M).

12. Is there an opportunity to participate in short-term clinical visit programs at your location? *Drop down menu.*

A.: Short-term clinical visit program of the young DEGRO. Brachy short-term clinical visit program of the young DEGRO. I am not familiar with the jDEGRO short-term clinical visit programs. Others.

13. Which rotation/short-term clinical visit is obligatory at your clinic? *Drop down menu.*

A.: To an external location of the same subject. At the same location in another subject.

At an external location in another subject. No obligatory rotation. Others.

14. How many (%) of the residents at your hospital take part in a short-term clinical visit/rotation program at least once during their training? *Drop down menu.*

A.: All. 75%. 50%. 25%. A few. None.

15. How many (%) of the residents at your hospital receive reimbursement for educational/webinar expenses? *Drop down menu. The questions should be answered separately in relation to training/webinars in Germany, in the EU and outside the EU.*

A.: All. 75%. 50%. 25%. A few. None. Not assessable.

16. How many (%) of the residents at your hospital should receive reimbursement for educational/webinar expenses? *Drop down menu. The questions should be answered separately in relation to training/webinars in Germany, in the EU and outside the EU.*

A.: All. 75%. 50%. 25%. A few. None. Not assessable.

17. At your hospital, how much of the residents are reimbursed for congresses?

Drop down menu. The questions should be answered separately in relation to congresses in Germany, in the EU and outside the EU.

A.: All. 75%. 50%. 25%. A few. None. Not assessable.

18. At your hospital, how many percent of the residents should be reimbursed for congresses? *Drop down menu. The questions should be answered separately in relation to congresses in Germany, in the EU and outside the EU.*

A.: All. 75%. 50%. 25%. A few. None. Not assessable.

19. Will your hospital reimburse the costs of a scientific congress (e.g. DEGRO annual meeting)? *Drop down menu.*

A.: Yes, in principle always. Yes, but only if you hold a lecture. Yes, but only if you present a lecture or a poster. No, in basically not.

20. How many days (d) per year is time off for external training events possible? *Drop down menu. The question should be answered separately in relation to preferred frequency vs. actual frequency.*

A.: 0d. 1–3d. 4–5d. > 5d. Not assessable.

21. On average, what percentage of the costs do you get reimbursed for training/webinars or congresses? *Drop down menu. The question should be answered separately in relation to* training/webinars or congresses.

A.: 0%. 25%. 50%. 75%. 100%. Not assessable.

22. On average, which percentage of the costs should be reimbursed for further training/webinars or congresses? *Drop down menu. The question should be answered separately in relation to* training/webinars or congresses.

A.: 0%. 25%. 50%. 75%. 100%. Not assessable.

23. Are the costs of mandatory courses in residency training (e.g., radiation protection training) covered? *Drop down menu.*

A.: Yes, always and in total. Yes, but only on a proportionate basis. No.

24. Do all colleagues in your workplace have the same opportunity to participate in training, etc., regardless of their gender, religion or sexual orientation? *Drop down menu.*

A.: Yes. No. Do not know.

25. Which of the following forms of knowledge transfer are available at your site? *Drop down menu.*

A.: Annual continuing education dialog with training officers. Written orientation concepts for the respective workplace. Mentoring discussions. Internal manual from assistant physicians for assistant physicians. Assessment of own level of knowledge at the beginning of a rotation with the responsible superiors. Assessment of one’s own level of knowledge at the end of a rotation with the responsible superiors. Participation in tumor boards. Learning-by-doing. Other.

26. Which of the following forms of knowledge transfer should be available at your site? *Drop down menu.*

A.: Annual continuing education dialog with training officers. Written orientation concepts for the respective workplace. Mentoring discussions. Internal manual from assistant physicians for assistant physicians. Assessment of own level of knowledge at the beginning of a rotation with the responsible superiors. Assessment of one’s own level of knowledge at the end of a rotation with the responsible superiors. Participation in tumor boards. Learning-by-doing. Other.

27. In your opinion, how important are the following forms of knowledge transfer? Arrange from important to not important (1 to 8). *Drop down menu with a range from 1 to 8.*

A.: Annual continuing education dialog with training officers. Written orientation concepts for the respective workplace. Mentoring discussions. Internal manual from assistant physicians for assistant physicians. Assessment of own level of knowledge at the beginning of a rotation with the responsible superiors. Assessment of one’s own level of knowledge at the end of a rotation with the responsible superiors. Participation in tumor boards. Learning-by-doing.

28. Who typically represents the discipline of radiation oncology on tumor boards at your site? *Drop down menu.*

A.: Assistant physician:in accordance with specialist guidance. Specialist physicians. Senior physician. Chief physician. There are no tumor boards available at my site.

29. Are you aware of what is expected in terms of competencies within residency training leading up to the specialist exam? *Drop down menu.*

A.: Yes. No.

30. Are you familiar with the topics of the curriculum of the working group *DEGRO Academy*? *Drop down menu.*

A.: Yes. No.

31. Are you aware of what is asked or expected of you in the residency exam? *Drop down menu.*

A.: Yes. No.

32. At your site, do you feel adequately prepared for the residency exam? Arrange from very unsatisfied to very satisfied (1 to 10). *Drop down menu with a range from 1 to 10.*
33. How well would you rate your knowledge of the following topics? Arrange from very bad to very good (1 to 10). *Drop down menu with a range from 1 to 10.*

A.: Breast cancer, Prostate cancer, Lung cancer, Colorectal cancer, Gynecological cancers (except breast), Head and neck tumors, Lymphoma, Skin tumors, Diseases of urinary organs, Brain metastases, Brain tumors.

34. How important are training opportunities on the following topics to you? Arrange from not very important to very important (1 to 10). *Drop down menu with a range from 1 to 10.*

A.: Breast cancer, Prostate cancer, Lung cancer, Colorectal cancer, Gynecological cancers (except breast), Head and neck tumors, Lymphoma, Skin tumors, Diseases of urinary organs, Brain metastases, Brain tumors.

35. Overall, how satisfied are you with your residency training? Arrange from very unsatisfied to very satisfied (1 to 10). *Drop down menu with a range from 1 to 10.*
36. In your view, should yDEGRO support a uniform, location-independent further education curriculum in Germany? *Drop down menu.*

A.: Yes. No.

37. What are your expectations and wishes for radiotherapy residency training in Germany?

A.: *optional free text-answers.*
